# Clinical Manifestations and Outcomes in Adults Hospitalized With Respiratory Syncytial Virus and Influenza a/B: A Multicenter Observational Cohort Study

**DOI:** 10.1093/ofid/ofae513

**Published:** 2024-09-20

**Authors:** Clara Lundetoft Clausen, Amanda Marie Egeskov-Cavling, Noor Hayder, Adin Sejdic, Casper Roed, Jon Gitz Holler, Lene Nielsen, Mads Frederik Eiberg, Omid Rezahosseini, Christian Østergaard, Zitta Barrella Harboe, Thea K Fischer, Thomas Benfield, Birgitte Lindegaard

**Affiliations:** Center of Research and Disruption of Infectious Diseases (CREDID), Department of Infectious Diseases, Amager and Hvidovre Hospital, Copenhagen, Denmark; Department of Clinical Research, Copenhagen University Hospital, North Zealand Hospital, Hilleroed, Denmark; Center of Research and Disruption of Infectious Diseases (CREDID), Department of Infectious Diseases, Amager and Hvidovre Hospital, Copenhagen, Denmark; Department of Pulmonary and Infectious Diseases, Copenhagen University Hospital, North Zealand Hospital, Hilleroed, Denmark; Department of Pulmonary and Infectious Diseases, Copenhagen University Hospital, North Zealand Hospital, Hilleroed, Denmark; Department of Pulmonary and Infectious Diseases, Copenhagen University Hospital, North Zealand Hospital, Hilleroed, Denmark; Department of Clinical Medicine, Faculty of Health and Medical Science, University of Copenhagen, Copenhagen, Denmark; Department of Pulmonary and Infectious Diseases, Copenhagen University Hospital, North Zealand Hospital, Hilleroed, Denmark; Department of Clinical Microbiology, Herlev and Gentofte Hospital, Copenhagen, Denmark; Department of Pulmonary and Infectious Diseases, Copenhagen University Hospital, North Zealand Hospital, Hilleroed, Denmark; Department of Pulmonary and Infectious Diseases, Copenhagen University Hospital, North Zealand Hospital, Hilleroed, Denmark; Diagnostic Infectious Disease Preparedness, Statens Serum Institut, Copenhagen, Denmark; Department of Pulmonary and Infectious Diseases, Copenhagen University Hospital, North Zealand Hospital, Hilleroed, Denmark; Department of Clinical Medicine, Faculty of Health and Medical Science, University of Copenhagen, Copenhagen, Denmark; Department of Clinical Research, Copenhagen University Hospital, North Zealand Hospital, Hilleroed, Denmark; Department of Public Health, University of Copenhagen, Copenhagen, Denmark; Center of Research and Disruption of Infectious Diseases (CREDID), Department of Infectious Diseases, Amager and Hvidovre Hospital, Copenhagen, Denmark; Department of Pulmonary and Infectious Diseases, Copenhagen University Hospital, North Zealand Hospital, Hilleroed, Denmark; Department of Clinical Medicine, Faculty of Health and Medical Science, University of Copenhagen, Copenhagen, Denmark

**Keywords:** respiratory syncytial virus, mortality, adults, clinical epidemiology, respiratory infections

## Abstract

**Background:**

Respiratory syncytial virus (RSV) and influenza cause significant health challenges, particularly for individuals with comorbid conditions and older adults. However, information on the clinical manifestations and outcomes of adults hospitalized with RSV in Europe remains limited.

**Methods:**

This multicenter observational cohort study of adults hospitalized with RSV or influenza A or B from March 2016 to April 2020 investigated the clinical manifestations, mortality risk factors, and association with 90-day mortality rates by logistic regression analysis after adjustment for covariates.

**Results:**

Of 988 patients hospitalized with either virus, 353 had RSV, 347 had influenza A, and 288 had influenza B infection. Patients with RSV, compared with those with influenza A or B, were more likely to have comorbid conditions (83% for RSV vs 72% for influenza A [*P* = .03] and 74% for influenza B [*P* = .001]) or pneumonia (41% vs 29% [*P* = .03] and 24% [*P* < .001], respectively). After adjustment for covariates, RSV infection was associated with an increased all-cause mortality rate within 90 days compared with influenza B (odds ratio, 2.16 [95% confidence interval, 1.20–3.87]; *P* = .01) but not influenza A (1.38 [.84–2.29]; *P* = .21). Increasing age and present pneumonia were identified as independent mortality risk factors in patients with RSV.

**Conclusions:**

Older adults hospitalized with RSV infections are at a higher risk of dying within 90 days of hospitalization than patients admitted with influenza B but at a similar risk as those admitted with influenza A, emphasizing the detrimental effects and severity of older patients being infected with RSV. Our findings underscore the need for strategic testing and vaccination approaches to mitigate the impact of RSV among older adults.

In recent years, respiratory syncytial virus (RSV) has been acknowledged to cause severe acute respiratory infections in adults [1, 2]. In the European Union, an estimated 158 229 RSV-associated hospitalizations occur annually among adults [3]. RSV infection in adults is more severe among older individuals and those with underlying comorbid conditions such as chronic obstructive pulmonary disease (COPD) and immunodeficiency, predisposing to increased disease severity and unfavorable outcomes [4, 5].

Clinical manifestations of RSV in adults range from mild coldlike symptoms to severe respiratory distress [5]. Recent studies from the United States and Israel have shown that RSV-related hospitalizations are less frequent than influenza but that the severity of RSV infection is comparable to that of influenza, if not more severe [6, 7]. However, within the European continent, limited data exist on RSV-associated hospitalizations among adults. Further information on RSV-related outcomes and patients at risk is required for policymaking, particularly related to in-hospital testing strategy and in the context of RSV vaccination strategies. This study aimed to examine the clinical characteristics and manifestations of patients hospitalized with RSV, compared with those in patients hospitalized with influenza A or B; to evaluate disease severity and mortality differences between patients with RSV and those with influenza A or B; and to assess risk factors for mortality in patients with RSV and those with influenza A or B.

## METHODS

### Study Design and Participants

This is a multicenter, retrospective cohort study of patients hospitalized with RSV, influenza A, or influenza B. Patients were hospitalized at Copenhagen University Hospital, Amager and Hvidovre, and Copenhagen University Hospital, North Zealand, from March 2016 through April 2020. Inclusion criteria comprised all adult patients (aged ≥18 years) with a positive test result for either RSV or influenza A or B within 48 hours of admission, who were hospitalized for ≥12 hours. The tests included oropharyngeal or nasal swab, sputum, endotracheal aspirate, or bronchoalveolar lavage samples. Exclusion criteria were dual or triple infection with RSV, influenza A, or influenza B. The study was reported according to the STROBE statement [8].

### Ethics and Patient Consent Statement

The study was approved by the Danish Health and Medicines Authority (R-20062127) and by the Danish Data Protection Agency (J-23065923), which gave administrative permission to access raw anonymized data used in the study. By Danish law, observational studies performed in Denmark do not need approval from a medical ethics committee.

### Data Collection

Data was manually extracted by reviewing electronic health records, including demographic variables, comorbid conditions, duration and nature of symptoms, vital parameters (heart rate, peripheral oxygen saturation, temperature, systolic and diastolic blood pressure, respiratory rate), treatment limitations (restrictions from intensive care unit [ICU] admission and cardiopulmonary resuscitation), bacterial coinfection in the bloodstream, positive lower respiratory tract samples, and clinical outcome (admission to ICU, use of mechanical ventilation, length of stay, readmission, and/or death). Paraclinical data included biochemical parameters within the first day of admission (plasma C-reactive protein, lactate dehydrogenase, creatinine, and alanine aminotransferase as well as blood leukocyte count, neutrophil, lymphocyte, and platelet counts), microbiological parameters (sputum, blood) during admission, and radiological parameters (chest radiograph) at admission. Biochemical data was retrieved from the Department of Biochemistry within each hospital. Pneumonia was defined as the presence of a new-onset infiltrate on a chest radiograph assessed by a radiologist and presenting symptoms or clinical signs consistent with pneumonia, such as cough, dyspnea, wheezing, and fever. The data were entered and stored in a Research Electronic Data Capture (REDCap) database [9].

### Viral Respiratory Analysis

Alere i Influenza A *&* B and Alere i RSV (Abbott Laboratories) and cobas Influenza A/B & RSV (Roche Diagnostics, F. Hoffmann-La Roche) assays were used. All samples were verified with the reference method (Prodesse ProFlu+ Assay [influenza A/B and RSV]; Hologic), performed at the Department of Clinical Microbiology, Herlev and Hvidovre Hospital.

### Statistical Analysis

Normally distributed continuous variables were presented as means and standard deviations, and nonnormally distributed continuous variables as medians and interquartile ranges (IQRs). Comparison of demographic variables and clinical characteristics between patients with RSV, influenza A, and influenza B were performed using χ^2^ tests, 1-way analysis of variance, and Kruskal-Wallis tests, as appropriate. Categorical data were reported as frequency counts and percentages for each category, and χ^2^ tests were used to estimate the *P* values. Age was divided into 4 groups: 18–49, 50–64, 65–79, and >80 years. Kaplan-Meier plots were performed to display survival data with a log-rank test for 90-day mortality. Univariable and multivariable logistic regression models were performed to calculate the odds ratios (ORs) with 95% confidence interval (CIs) for death during hospitalization or within 90 days in patients admitted with RSV, compared with those admitted with influenza A or B infections. Adjusted parameters included age, sex, comorbid conditions (hypertension, diabetes, COPD, asthma, congestive heart failure [CHF], acute myocardial infarction [AMI], and cancer), pneumonia, year of testing, positive lower respiratory tract sample, and bacteremia.

A spline function for age was incorporated into the models. Splines provide a flexible approach to modeling age, accommodating potential nonlinear relationships, and allowing for a more accurate representation of its effect on mortality risk. *P* values <.05 were considered statistically significant. A deviance goodness-of-fit test was applied for model control in the univariable models. Competing risk analysis using cumulative incidence function within each group was performed for ICU admission and readmission within 90 days. Comparisons were performed using Gray's test. Univariable and multivariable logistic regression analyses were applied to estimate ORs with 95% CIs for risk factors associated with death among patients with RSV, influenza A, or influenza B. A spline function for age was also included in the models. Initially, univariable models were used to identify potential risk factors, selecting variables with *P* values <.10 for further consideration.

Subsequently, all identified risk factors from the univariable analyses were included in the multivariable models for each pathogen. To account for type I errors, the Holm method was applied to correct *P* values for pairwise and multiple comparisons. To avoid selection bias, we performed a sensitivity analysis by hospital site, presence of pneumonia, ≥2 respiratory symptoms, and presence of a diagnostic test result within 24 hours of admission. We also performed a sensitivity analysis of the multivariate logistic regression model, including treatment restrictions. All statistical analyses were carried out using R statistical software, version 4.3.0 [10].

## RESULTS

### Characteristics

A total of 1150 hospitalized individuals were identified within the 2 study sites during the study period. Individuals with dual RSV and influenza (n = 36), individuals admitted for <12 hours (n = 73), and individuals who tested positive ≥48 hours after admission (n = 53) were excluded (n = 162 in total), yielding a final cohort of 988 patients. Of these, 353 (36%) were hospitalized with RSV, 347 (35%) with influenza A, and 288 (29%) with influenza B. Baseline characteristics are provided in Table 1.

**Table 1.** Demographic and Clinical Characteristics of Patients With Respiratory Syncytial Virus, Influenza A, or Influenza B Infection

Characteristic	Patients, No. (%)^a^		
	RSV (n = 353)	Influenza A (n = 347)	Influenza B (n = 288)
Female sex	192 (54)	178 (51)	163 (57)
Age, median (IQR), y	74 (63–83)	69 (53–81)	73 (61–82)
Age group			
18–49 y	40 (11)	83 (24)	33 (12)
50–64 y	56 (16)	68 (20)	60 (21)
65–79 y	137 (39)	100 (29)	99 (34)
>80 y	120 (34)	96 (28)	96 (33)
Comorbid conditions			
Hypertension	171 (48)	144 (41)	129 (45)
CHF	126 (36)	54 (16)	116 (40)
Previous AMI	51 (14)	38 (11)	49 (17)
Asthma	62 (17)	53 (15)	43 (15)
COPD	120 (34)	82 (24)	68 (24)
Previous or current cancer	82 (23)	47 (14)	55 (19)
Diabetes	60 (17)	54 (16)	57 (20)
Pneumonia^b^	143 (41)	100 (29)	70 (24)
No. of comorbid conditions			
0	61 (17)	97 (28)	76 (26)
1	91 (26)	120 (35)	59 (21)
2	80 (23)	71 (20)	51 (18)
≥3	119 (33.4)	60 (17.1)	102 (35.1)
Smoking status			
Never	90 (26)	99 (29)	49 (17)
Former	171 (48)	131 (38)	110 (38)
Current	65 (18)	67 (19)	25 (9)
Unknown	25 (7)	50 (14)	104 (36)
Alcohol use			
None	101 (29)	132 (38)	89 (31)
Moderate use (<14 units/wk^c^)	124 (35)	111 (32)	75 (26)
Overconsumption (>14 units/wk^c^)	21 (6)	20 (6)	11 (4)
Unknown	107 (30)	84 (24)	113 (39)
Paraclinical parameters, median (IQR)			
Plasma CRP, mg/L	29 (11–65)	49 (22–98)	25 (8–74)
Plasma ALT, U/L	21 (14–31)	23 (17–35)	20 (13–29)
Plasma LDH, U/L	210 (176–244)	210 (182–260)	200 (171–236)
Blood leukocyte count, ×10^9^/L	9.1 (6.9–11.8)	7.9 (6.0–10.8)	8.2 (5.7–11.5)
Blood neutrophil count, ×10^9^/L	6.3 (4.5–9.0)	5.8 (4.2–8.0)	5.3 (3.4–8.5)
Blood lymphocyte count ×10^9^/L	1.4 (0.9–1.9)	0.9 (0.6–1.5)	1.3 (0.9–1.9)
Blood platelet, count, ×10^9^/L	252 (186–326)	209 (159–268)	230 (178–312)
Plasma creatinine, μmol/L	76 (60–96)	78 (64–99)	79 (64–106)
eGFR, mL/min/1.73m^2^	58 (23–77)	63 (39–79)	57 (15–75)
Infiltrate on chest radiograph	155 (44)	105 (30)	78 (27)
Bacteremia	9 (2.5)	15 (4.3)	9 (3.1)
Positive bacterial LRTS	54 (15)	38 (11)	29 (10)
Vital parameters at admission, median (IQR)			
Respiratory, breaths per minute	20 (18­–24)	20 (18–22)	20 (16–22)
Peripheral oxygen saturation, %	95 (94–97)	96 (94–98)	96 (94–98)
Heart rate, beats per minute	92 (79–108)	93 (80–110)	86 (74–97)
Systolic BP, mm Hg	137 (121–155)	132 (120–146)	129 (114–151)
Diastolic BP, mm Hg	77 (67–86)	74 (65–83)	70 (63–81)
Temperature, °C	37.4 (36.8–38.1)	37.6 (36.8–38.5)	37.8 (37.1–38.5)
Symptoms			
Fever	164 (47)	194 (56)	145 (50)
Dyspnea	226 (64)	176 (51)	113 (39)
Throat pain	30 (9)	28 (8)	7 (2)
Cough	275 (78)	271 (78)	199 (69)
Headache	36 (10)	84 (24)	46 (16)
Diarrhea	11 (3)	39 (11)	30 (10)
Nausea	30 (9)	84 (24)	49 (17)
Fatigue	71 (20)	86 (25)	49 (17)
Wheezing	2 (0.6)	5 (1.4)	1 (0.3)
Duration of symptoms			
Median (IQR), d	4 (2–7)	3 (1–6)	4 (2–7)
Data missing, no.	78	31	68

Abbreviations: ALT, alanine aminotransferase; AMI, acute myocardial infarction; BP, blood pressure; CHF, congestive heart failure; COPD, chronic obstructive pulmonary disease; CPAP, continuous positive airway pressure; CRP, C-reactive protein; eGFR, estimated glomerular filtration rate; ICU, intensive care unit; IQR, interquartile range; LDH, lactate dehydrogenase; LMWH, low molecular weight heparin; LRTS, lower respiratory tract sample; NIV, noninvasive ventilation; NOACS, non–vitamin K antagonist oral anticoagulants; RSV, respiratory syncytial virus.

^a^Data represent no. (%) of patients unless otherwise specified.

^b^Pneumonia is defined as a new infiltrate on chest radiograph described by a radiologist and ≥1 respiratory symptom, such as cough, wheezing, dyspnea, throat pain, or fever.

^c^One unit was defined as 12 g of alcohol.

We found that patients hospitalized with RSV were of the same median age as those with influenza B, while both were older than patients with influenza A (Table 1). Patients with RSV more often had ≥1 comorbid condition (82%) than those with influenza A (72%; *P* = .03) or influenza B (74%; *P* = .007). Overall, hypertension was the most common, followed by CHF and COPD. Patients with RSV were observed to have CHF (36%) and COPD (34%) more frequently than those with influenza A (16% [*P* = .003] and 24% [*P* = .007], respectively) and at proportions comparable to or higher than patients with influenza B (40% [*P* = .27] and 24% [*P* = .009], respectively). Pneumonia was detected in almost half of the patients with RSV (41%), compared with less than a third in influenza A or B (29% [*P* = .003] and 24% [*P* < .001], respectively). Among patients with pneumonia, a positive lower respiratory tract sample was present in 8% of those with RSV, slightly higher than in influenza A at 5% (*P* = .21) and influenza B at 4% (*P* = .10).

Patients with influenza A or B had slightly higher median body temperatures on admission, 37.6°C (IQR, 36.8°C–38.5°C) and 37.8°C (37.1°C–38.5°C), respectively, compared with 37.4°C (36.8°C–38.1°C; *P* = .03 and *P* < .001, respectively) in patients with RSV. Levels of C-reactive protein at admission were higher in patients with influenza A (median [IQR], 49 [22–98] mg/L) than in those with RSV (29 [11–65] mg/L; *P* = .003) or influenza B (25 [8–74] mg/L; *P* = .003). Respiratory symptoms, including dyspnea, cough, and fever were the most common symptoms in all groups. Headache, nausea, and diarrhea were more frequently reported in patients with influenza A and B than in those with RSV. Dyspnea was more prevalent in patients with RSV (64%) than in those with influenza A (51%) or B (39%) (both *P* = .003).

### Clinical Outcomes

Clinical outcomes are provided in Table 2. During hospitalization, 50 patients (5%) were admitted to the ICU, with no differences between the groups. Of these, 16 (1.6%) received invasive mechanical ventilation. Close to a third of patients with RSV 97 (28%) were under treatment restrictions and not considered candidates for invasive mechanical ventilation in the ICU, compared with 40 (12%) with influenza A and 5 (2%) with influenza B (both *P* < .001). A total of 49 patients (5%) died during hospitalization, with no differences between the groups. In total, 209 patients (21%) were readmitted within 90 days. There were no differences in readmissions between the 3 groups (Supplementary Figures 1 and 2).

**Table 2.** Clinical Outcomes in Patients With Respiratory Syncytial Virus, Influenza A, or Influenza B

Outcome	Patients, No. (%)^a^		
	RSV (n = 353)	Influenza A (n = 347)	Influenza B (n = 288)
Length of stay, median (IQR), d	3 (1–7)	3 (1–6)	3 (1–6)
Oxygen required during hospitalization	203 (58)	206 (59)	142 (49)
ICU admission	18 (5)	20 (6)	12 (4)
Treatment limitation			
IMV treatment in the ICU	97 (28)	40 (12)	5 (2)
No CPR	101 (29)	43 (12)	6 (2)
Intubation	3 (0.8)	9 (2.6)	4 (1.4)
Readmission^b^	82 (23)	67 (19)	60 (21)
Deaths			
In-hospital	26 (7.4)	14 (4.0)	9 (3.1)
Within 30 d	35 (9.9)	23 (6.6)	17 (5.9)
Within 90 d	61 (17.3)	32 (9.2)	22 (7.6)
Within 180 d	72 (20.4)	39 (11.2)	28 (9.7)

Abbreviations: CPR, cardiopulmonary resuscitation; ICU, intensive care unit; IMV, invasive mechanical ventilation; IQR, interquartile range; RSV, respiratory syncytial virus.

^a^Data represent no. (%) of patients unless otherwise specified.

^b^Readmission within 90 days after admission.

**Figure 1.**
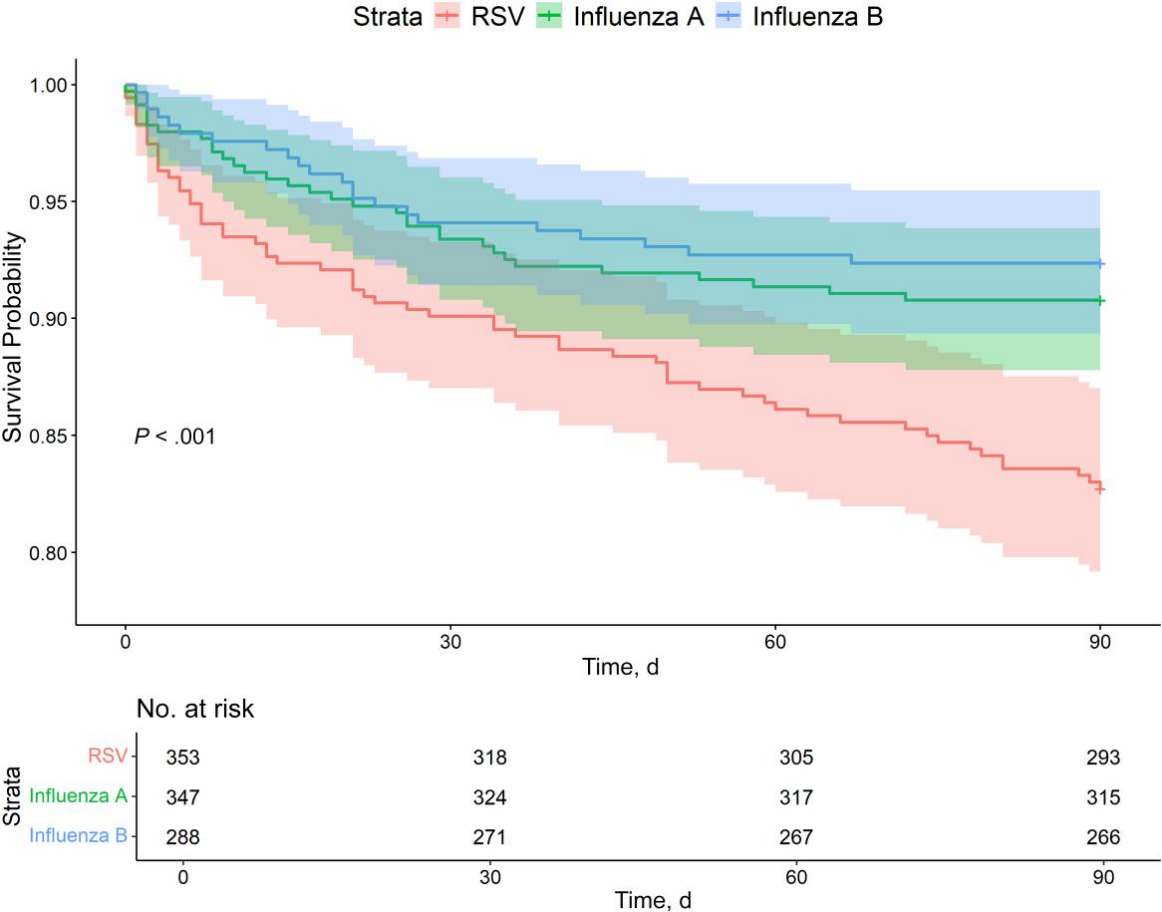
Kaplan-Meier survival curve of the 90-day mortality rate in patients with respiratory syncytial virus (RSV), influenza A, or influenza B. *P* values were estimated using a log-rank test.

**Figure 2.**
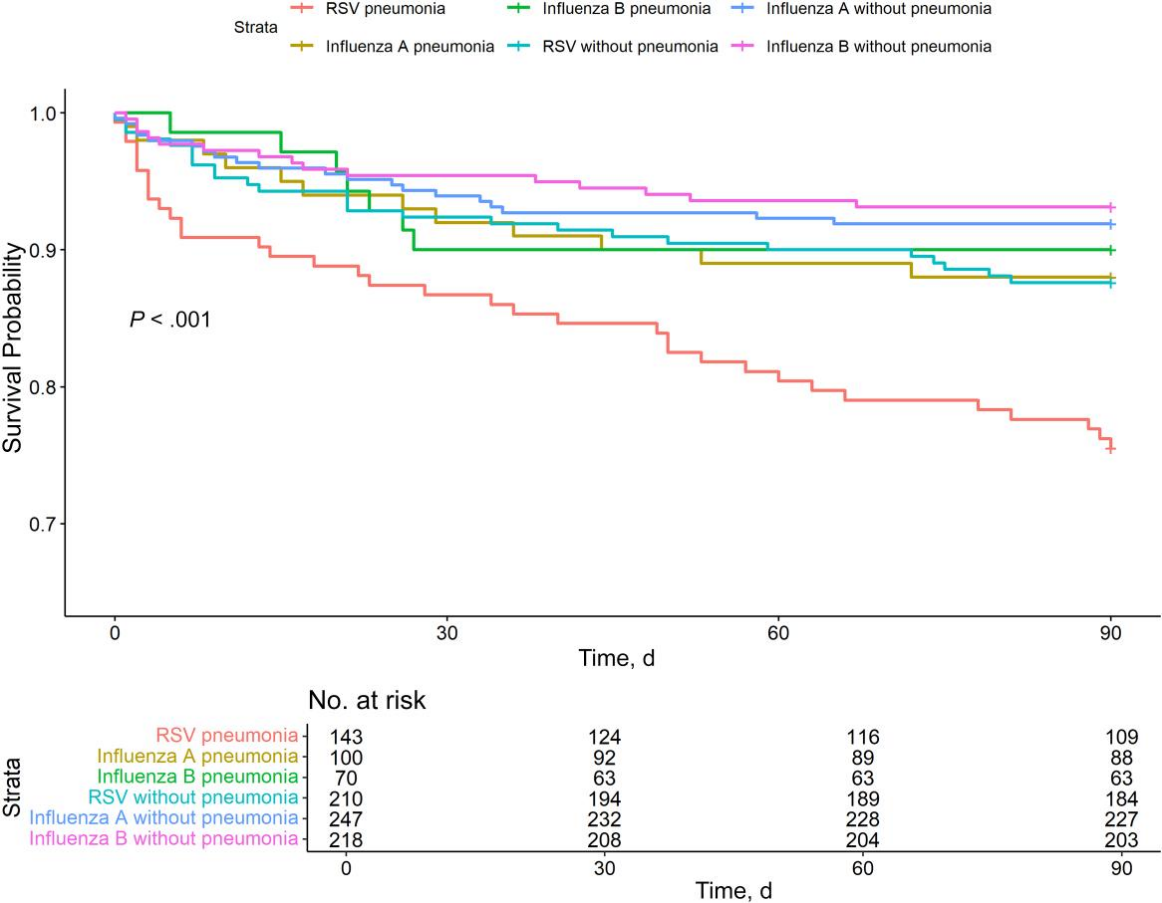
Kaplan-Meier survival curve of 90-day mortality rate in patients with respiratory syncytial virus (RSV), influenza A, or influenza B, stratified into those with and those without radiographically confirmed pneumonia. *P* values were estimated using a log-rank test.

A significantly higher proportion of patients with RSV died within 90 days (17%), compared with the proportions for influenza A (9%; *P* = .005) and influenza B (8%; *P* = .001) (Table 2 and Figure 1). Similar mortality rates were observed when the analysis was restricted to those with pneumonia (Figure 2). Crude and adjusted logistic regression models for associations between in-hospital and 90-day mortality rates and infection with RSV, influenza A, and influenza B are presented in Table 3. RSV was associated with higher odds of death within 90 days in the unadjusted model, compared with both influenza A (OR, 2.06 [95% CI, 1.30–3.25]; *P* = .002) and influenza B (2.53 [1.51–4.23]; *P* < .001) (Table 3). After adjustment for covariates, the association remained significantly higher than for influenza B (OR, 2.16 [95% CI, 1.2–3.87]; *P* = .01) but not influenza A (1.38 [.84–2.29]; *P* = .21).

**Table 3.** Crude and Adjusted Logistic Regression Analysis of Associations Between Mortality and Hospitalization With Respiratory Syncytial Virus, Influenza A, or Influenza B

Mortality Risk	Univariable OR (95% CI)	*P* Value	Multivariable OR (95% CI)^a^	*P* Value
In-hospital mortality				
Influenza A (reference)	1	…	1	…
RSV	1.89 (.97–3.69)	.06	1.55 (.76–3.16)	.23
Influenza B	.77 (.33–1.80)	.54	.69 (.27–1.75)	.44
Influenza B (reference)	1	…	1	…
RSV	2.46 (1.14–5.35)	.02^b^	2.23 (.95–5.27)	.07
90-d mortality				
Influenza A (reference)	1	…	1	…
RSV	2.06 (1.30–3.25)	.002^b^	1.38 (.84–2.29)	.21
Influenza B	.81 (.46–1.44)	.48	.64 (.34–1.21)	.17
Influenza B (reference)	1	…	1	…
RSV	2.53 (1.51–4.23)	<.001^b^	2.16 (1.2–3.87)	.01^b^

Abbreviations: CI, confidence interval; OR, odds ratio; RSV, respiratory syncytial virus.

^a^Adjusted for age, sex, hypertension, congestive heart failure, diabetes, cancer, chronic obstructive pulmonary disease, asthma, pneumonia, and year of testing.

^b^Significant at *P* < .05.

There were no differences in 90-day mortality data between patients with influenza A and those with influenza B in crude and adjusted models. In crude models, the odds of in-hospital death were higher in patients with RSV than in those with influenza B (OR, 2.46 [95% CI, 1.14–5.35]; *P* = .02). However, the association did not remain statistically significant after adjustment (OR, 2.23 [95% CI, .95–5.27]; *P* = .06).

### Risk Factors for 90-Day All-Cause Mortality Rate

Table 4 presents the results from univariable and multivariable logistic regression analysis, identifying risk factors for the 90-day mortality rate. Age was modeled using a spline function to capture potential nonlinearity in both analyses. For patients with RSV, the univariable model revealed a nonlinear effect of age on mortality risk (effective degrees of freedom [edf] = 2.29; *P* < .001), indicating that mortality risk increases with age but varies across different age groups (see Supplementary Figure 3). In addition, the presence of pneumonia and prior AMI were associated with increased mortality risk, while asthma was a protective factor. For influenza A, age showed a nearly linear relationship with mortality risk in the univariable model. For patients with influenza A, COPD was also a significant risk factor. Finally, in patients with influenza B, we did not find any significant mortality risk factor. For the multivariable logistic regression analysis, age was a significant risk factor across all pathogens. For patients with RSV, the relationship between age and mortality became linear after adjustment (edf = 1; *P* = .001) (Supplementary Figure 3). In addition, having pneumonia remained an independent mortality risk factor and asthma a protective risk factor. For both patients with influenza A and those with B, only age remained as a significant risk factor after adjustment. The results were consistent regardless of the inclusion of pneumonia in the models.

**Table 4.** Univariable and Multiple Logistic Regression Models of 90-Day Mortality Risk Factors in Patients With Respiratory Syncytial Virus, Influenza A, or Influenza B

Risk Factors by Infection Type	Univariable Model				Multivariable Model			
	OR (95% CI)	edf	χ^2^	*P* Value^a^	OR (95% CI)	edf	χ^2^	*P* Value
RSV								
Age (spline)^b^	…	2.29	19.09	.01^c^	…	1	10.3	.001^c^
COPD	1.43 (.81–2.51)	…	…	>.99	1.32 (.72–2.14)	…	…	.37
Pneumonia	2.29 (1.31–4.02)	…	…	.03^c^	2.01 (1.11–3.66)	…	…	.02^c^
Asthma	.20 (.06–.67)	…	…	.07^c^	.28 (.08–.96)	…	…	.04^c^
AMI	2.32 (1.18–4.57)	…	…	.10^c^	1.98 (.96–4.09)	…	…	.07
Cancer	1.39 (.85–2.28)	…	.32	…	…	…	…	…
Male sex	1.01 (.58–1.76)	…	…	>.99	…	…	…	…
CHF	1.55 (.88–2.71)	…	…	>.99	…	…	…	…
Diabetes	1.09 (.53–2.24)	…	…	>.99	…	…	…	…
Hypertension	.96 (.55–1.66)	…	…	>.99	…	…	…	…
Influenza A								
Age (spline)^b^	…	1.00	19.52	.01^c^	…	1	17.32	<.001^c^
COPD	2.84 (1.34–5.99)	…	…	.06^c^	1.78 (.81–3.95)	…	…	.15
Pneumonia	1.55 (.73–3.30)	…	…	>.99	1.65 (.73–3.75)	…	…	.23
Asthma	.35 (.08–1.49)	…	…	>.99	.46 (.10–2.11)	…	…	.31
AMI	2.05 (.78–5.35)	…	…	>.99	1.05 (.38–2.90)	…	…	.92
Cancer	1.92 (.78–4.74)	…	…	>.99	…	…	…	…
Male sex	1.05 (.51–2.18)	…	…	>.99	…	…	…	…
CHF	1.29 (.50–3.30)	…	…	>.99	…	…	…	…
Diabetes	1.01 (.37–2.75)	…	…	>.99	…	…	…	…
Hypertension	1.11 (.53–2.32)	…	…	>.99	…	…	…	…
Influenza B								
Age (spline)^b^	…	1.01	5.944	.16	…	1.25	6.74	.02^c^
COPD	1.57 (.61–4.02)	…	…	>.99	1.32 (.49–3.56)	…	…	.58
Pneumonia	1.50 (.59–3.85)	…	…	>.99	1.20 (.45–3.22)	…	…	.71
Asthma	1.76 (.61–5.07)	…	…	>.99	2.11 (.69–6.45)	…	…	.19
AMI	.47 (.11–2.06)	…	…	>.99	.38 (.08–1.73)	…	…	.21
Cancer	1.24 (.54–2.81)	…	…	>.99	…	…	…	…
Male sex	.73 (.30–1.79)	…	…	>.99	…	…	…	…
CHF	.67 (.27–1.70)	…	…	>.99	…	…	…	…
Diabetes	1.21 (.43–3.43)	…	…	>.99	…	…	…	…
Hypertension	.55 (.22–1.39)	…	…	>.99	…	…	…	…

Missing values in certain rows indicate variables that were not included in the spline or multivariable model, so certain statistics (eg, *χ*²) were not calculated for all variables. Abbreviations: AMI, acute myocardial infarction; CHF, congestive heart failure; CI, confidence interval; COPD, obstructive pulmonary disease; edf, effective degrees of freedom; OR, odds ratio; RSV, respiratory syncytial virus.

^a^The Holm method was applied to adjusted *P* values for pairwise and multiple comparisons.

^b^Age (spline) indicates the use of spline function to model the nonlinear effect between age and 90-day mortality risk.

^c^Significant at *P* < .05.

### Sensitivity Analysis

In the sensitivity analysis, which included subgroups based on hospital site, those with pneumonia, those with >2 respiratory symptoms, and those who underwent testing within 24 hours of admission, respectively, the results remained consistently robust. When treatment restrictions were included in the adjusted 90-day mortality analysis, the mortality odds for patients with RSV were similar to those in patients with influenza A (OR, 0.98 [95% CI, .57–1.67]; *P* = .93) while slightly higher for those with influenza B, although this difference was not statistically significant (1.30 [.68–2.48]; *P* = .42).

## DISCUSSION

From March 2016 to April 2020, this multicenter study was conducted to analyze and compare the clinical characteristics, outcomes, and mortality risk factors in adults hospitalized with RSV or influenza A/B. Our results revealed that adults hospitalized with RSV were twice as likely to die within 90 days as those hospitalized with influenza B. Outcomes for RSV were comparable to those for influenza A. Patients hospitalized with RSV had a higher prevalence of chronic conditions and had pneumonia more often than patients with influenza A or B. Moreover, patients with RSV and influenza B were slightly older than those with influenza A. Increasing age and having pneumonia were independent 90-day mortality risk factors in patients with RSV.

In this study, the all-cause mortality rate in patients with RSV was higher than in previous studies [11–13]. Within 90 days, 17% of the RSV population died, which was substantially higher than reported by Descamps et al [11] (13%) and Tseng et al [13] (12%). A US study reported higher odds of death within 1 year compared with influenza (adjusted OR, 1.3 [1.0–1.6]; *P* = .02) but not death within 6 months (1.2 [0.9–1.5]; *P* = .18) [12]. A study by Descamps et al [11] reported an adjusted OR of 1.5 (95% CI, 1.0–2.3), for RSV-related death within 90 days of discharge, compared with influenza (*P* = .059. Although the higher odds of death in patients with RSV were not statistically significant in the study by Descamps et al, this may be attributed to the smaller study population (n = 108) and fewer events (n = 14). Our study revealed a doubled mortality risk within 90 days in patients with RSV compared with influenza B in the adjusted model. The cutoff at 90 days was chosen to enhance the likelihood of including all deaths related to RSV and influenza while minimizing deaths from other causes. Other studies have mostly reported in-hospital or 30-day mortality rates with little to no mortality risk in patients with RSV compared with influenza [14–17]. In our study, RSV was not associated with a higher risk of in-hospital death than influenza A or B.

Almost a third of patients infected with RSV had treatment restrictions during their admission, limiting both ICU admissions and cardiac resuscitation, suggesting a frail population among patients with RSV. A study from the United States [18] found acute functional decline secondary to hospitalization with RSV, especially among patients requiring care before hospitalization. This decline may contribute to the postdischarge mortality risk in an already frail population. Our sensitivity analysis further supports this, indicating that the increased mortality risk in patients with RSV was mainly driven by individuals subject to treatment restrictions.

Pneumonia was detected in almost half of the patients with RSV (41%), compared with a quarter of patients with influenza A and B (29% and 24%, respectively). Other studies have reported infiltrates on radiographs in patients with RSV with prevalences comparable to that in our study, ranging between 46% and 53% [13, 19]. Pneumonia was identified as a risk factor for death within 90 days in patients with RSV, and we saw that patients with pneumonia and RSV were more likely to die than patients with influenza and pneumonia. However, the odds of death remained higher in patients with RSV, after adjustment for pneumonia.

In the current study, patients with RSV had more comorbid conditions, particularly related to the cardiopulmonary system. This aligns with findings of previous studies identifying increased prevalence of COPD, asthma, and CHF among hospitalized patients with RSV, compared with those with influenza [7, 12, 16, 20, 21]. More frequently, patients with RSV presented with symptoms such as dyspnea, cough, and fever, suggestive of a mono-organ disease. On the other hand, patients with influenza frequently reported additional symptoms such as nausea, headache, and diarrhea, indicating systemic involvement, along with elevated temperatures on admission. This is consistent with findings of other studies [11, 15, 22].

The rates of oxygen supplementation during hospitalization, ICU admissions, length of hospital stay, and readmissions were comparable among patients with RSV, influenza A, and B. This contrasts with findings from prior studies, which reported higher likelihoods of patients with RSV receiving oxygen supplementation and being admitted to the ICU [6, 12, 20]. However, in the current study, close to a third of patients infected with RSV were not considered candidates for invasive mechanical ventilation, thereby limiting the ICU admission rate.

We documented that age is a significant risk factor for death within 90 days among patients admitted with RSV, influenza A, or influenza B. The nonlinear effect of age on the 90-day mortality risk in patients with RSV in the univariable model suggests that certain age groups are at a disproportionately higher risk, potentially due to varying susceptibility and the presence of other underlying conditions. The shift to a linear relationship in the multivariable model indicates that, when accounting for other risk factors like pneumonia and AMI, the influence of age on mortality risk becomes more consistent across ages. The linear increase in mortality risk with age for influenza A and B observed both in the univariable and multivariable models aligns with existing literature, indicating that age alone is a strong and consistent predictor of mortality risk for these pathogens [23].

The strengths of the current study primarily reside in its large population, consisting of consecutive patients admitted with RSV or influenza in 2 university hospitals within Denmark. Furthermore, a notable strength lies within the stringent inclusion criteria, specifically the requirement for testing within 48 hours of admission. This criterion enhanced the likelihood that the patients included in this study were hospitalized due to the viruses under consideration, rather than merely coincidentally harboring the viruses during hospitalization.

This study was not without limitations. The variability in testing protocols across different hospital sites and years, coupled with the absence of definitive testing guidelines introduces potential sampling bias. However, to mitigate the influence of temporal differences, we incorporated the year of the test into the adjusted outcome analysis. In addition, as data was gathered from 2 different sites, there was a risk of information bias. Moreover, we did not collect data on specific immunosuppressive disorders and vaccination status, limiting our ability to address their impact and association with the clinical manifestations and outcomes. Finally, a large proportion of the information about influenza B came from 2the 017–2018 season, when the dominant virus was the Yamagata line of influenza B, which is rarely seen in such a prominent role in elderly individuals and thus not included in the vaccine [24]. Therefore, our study mainly compared RSV with the Yamagata line of influenza B.

Historically, RSV infection has not been regarded as severe a disease in adults as influenza, leading to inconsistent testing strategies for RSV. Our findings contribute to the mounting evidence suggesting that hospitalizations with RSV infections can be as severe and deadly as influenza, if not more so, especially among older and frail populations [6, 7, 12, 20]. Further recognition of RSV as an important pathogen in severe acute respiratory infections is pivotal for better testing strategies, targeted treatment, including antiviral trials, and discontinuation of antibiotics, leading to better antibiotic stewardship, and vaccination strategies.

In conclusion, acute respiratory infections induced by RSV significantly contribute to disease and death among older adults. This highlights the severity and detrimental effects of RSV, which are similar to or worse than those of influenza A and B. The results underscore the potential life-threatening nature of RSV. Harmonizing in-hospital testing strategies, increasing awareness, and advocating for targeted RSV vaccinating efforts among high-risk groups and older adults is important.

## Supplementary Material

ofae513_Supplementary_Data

## Supplementary Data

Supplementary materials are available at *Open Forum Infectious Diseases* online. Consisting of data provided by the authors to benefit the reader, the posted materials are not copyedited and are the sole responsibility of the authors, so questions or comments should be addressed to the corresponding author.
