# Supplementary figures and images for: Clinical Manifestations and Outcomes in Adults Hospitalized With Respiratory Syncytial Virus and Influenza a/B: A Multicenter Observational Cohort Study
Source: Open Forum Infect Dis. 2024 Sep 20;11(10):ofae513. doi: 10.1093/ofid/ofae513 (PMC11474596; doi:10.1093/ofid/ofae513)

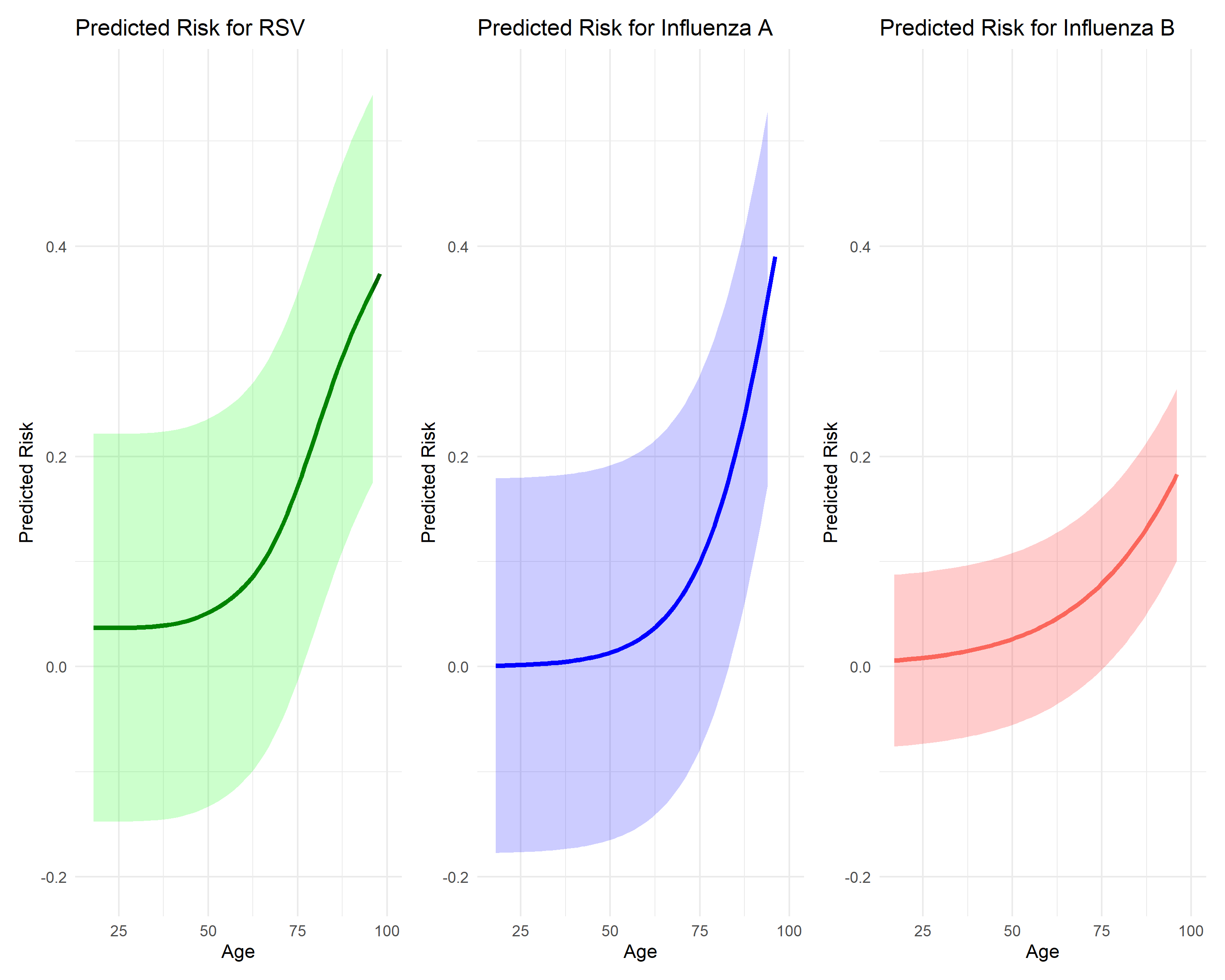

Supplement: ofae513_Supplementary_Data [file ofae513_supplementary_data.zip › supplementarty_Figure3.tiff]

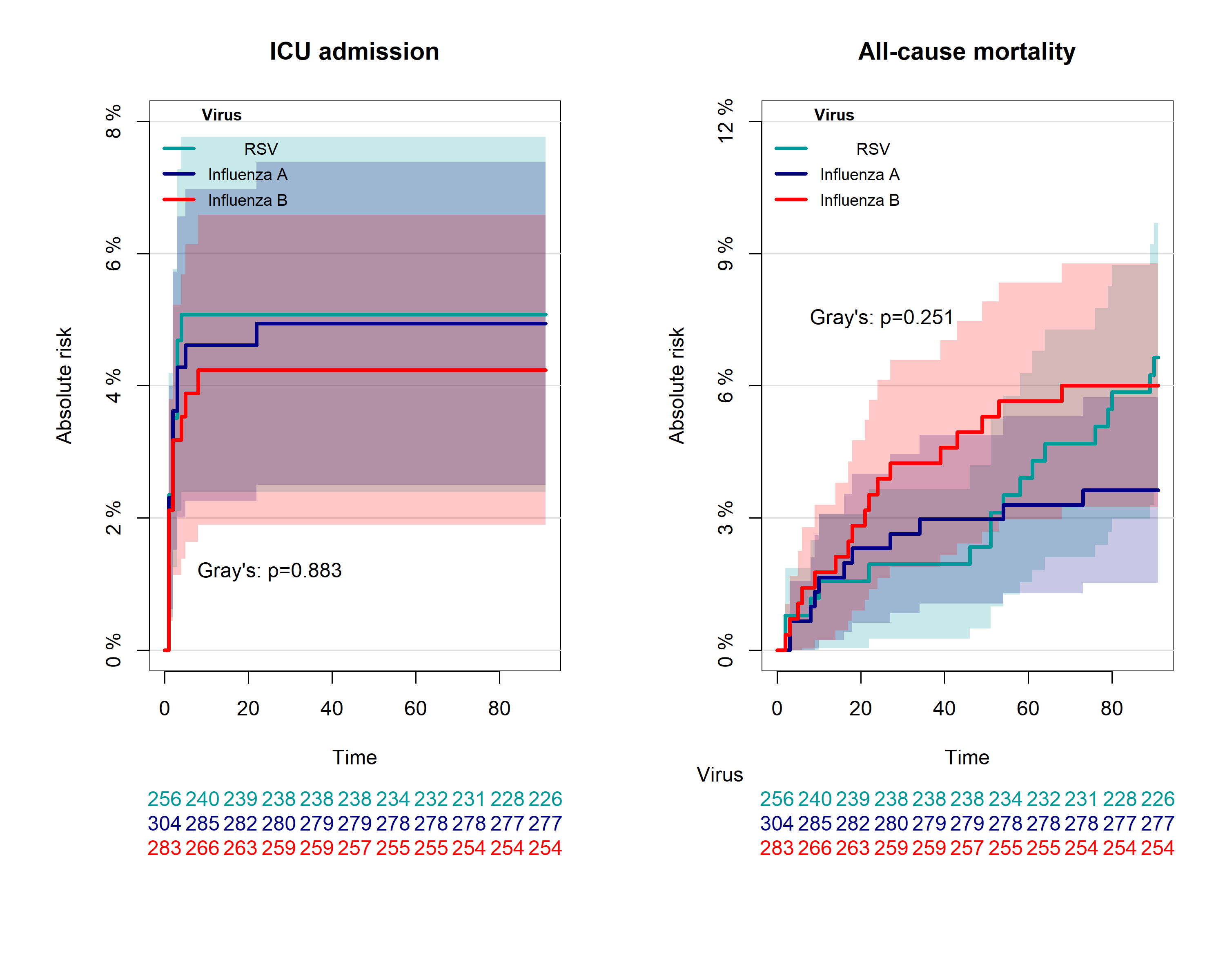

Supplement: ofae513_Supplementary_Data [file ofae513_supplementary_data.zip › Supplementary_figure1.tiff]

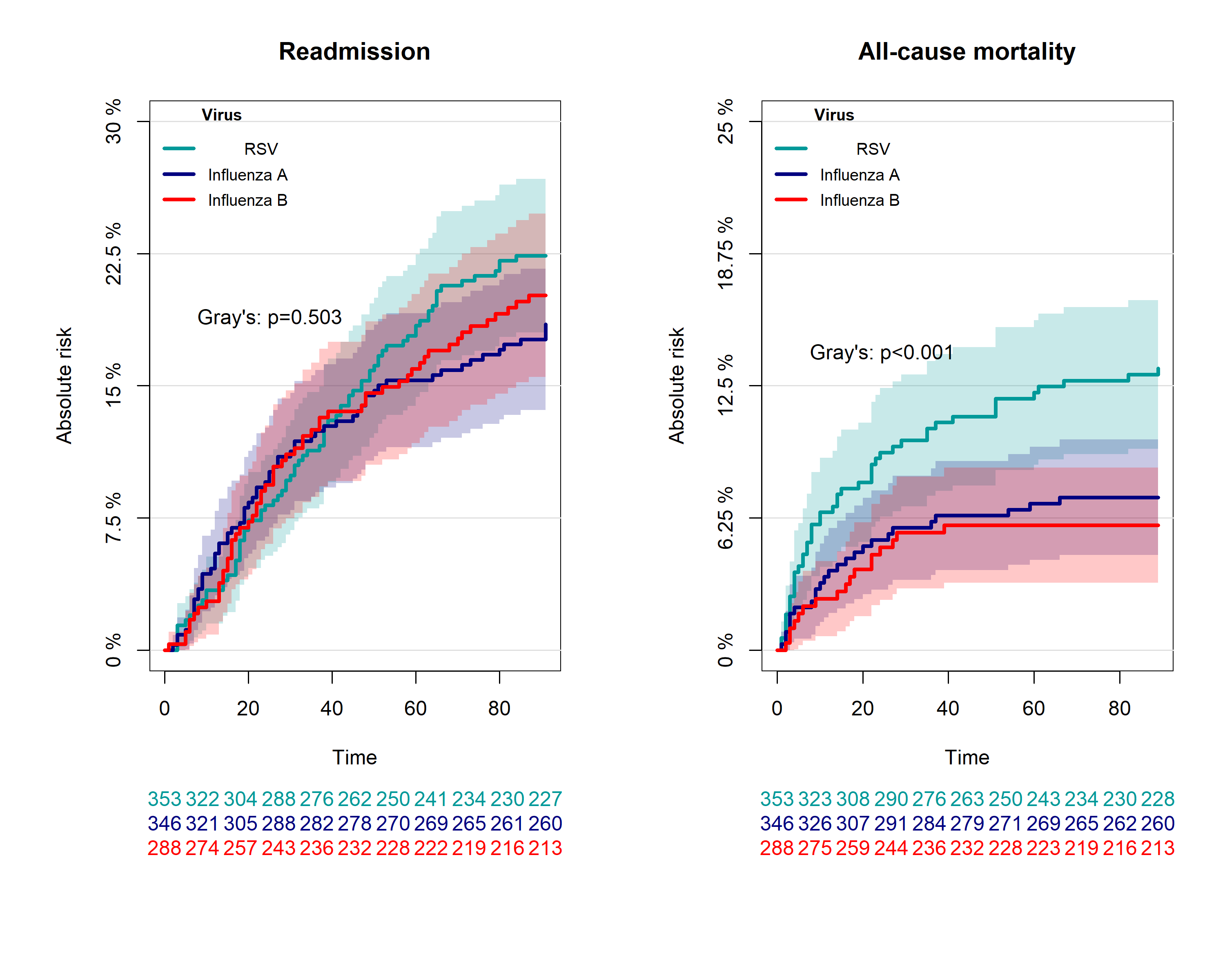

Supplement: ofae513_Supplementary_Data [file ofae513_supplementary_data.zip › Supplementary_figure2.tiff]
